# Age stratifies disease extent in pediatric and young adult fusion-positive papillary thyroid carcinoma

**DOI:** 10.1210/jendso/bvag184

**Published:** 2026-08-12

**Authors:** Sule Canberk, Zubair W Baloch, Anna Maria Carillo, Amber Isaza, Rita Barros, Mya Bojarsky, Andrew J Bauer

**Affiliations:** RISE-Health, Department of Pathology, Faculty of Medicine, University of Porto (FMUP), Porto 4200-319, Portugal; Department of Pathology and Laboratory Medicine, Children's Hospital of Philadelphia, University of Pennsylvania, Philadelphia, PA 19104, USA; RISE-Health, Department of Pathology, Faculty of Medicine, University of Porto (FMUP), Porto 4200-319, Portugal; Department of Public Health, University of Naples Federico II, Naples 80131, Italy; Pathology Unit, A.O.R.N. Cardarelli, Hospital Antonio Cardarelli, Naples 80131, Italy; Division of Endocrinology and Diabetes, The Thyroid Center, Children's Hospital of Philadelphia, Philadelphia, PA 19104, USA; RISE-Health, Department of Pathology, Faculty of Medicine, University of Porto (FMUP), Porto 4200-319, Portugal; Division of Endocrinology and Diabetes, The Thyroid Center, Children's Hospital of Philadelphia, Philadelphia, PA 19104, USA; Division of Endocrinology and Diabetes, The Thyroid Center, Children's Hospital of Philadelphia, Philadelphia, PA 19104, USA; Department of Paediatrics, Children's Hospital of Philadelphia, Perelman School of Medicine, Philadelphia, PA 19104, USA

**Keywords:** papillary thyroid carcinoma, pediatric thyroid cancer, kinase fusions, *RET*, *NTRK*, molecular risk stratification

## Abstract

**Context:**

Fusion-positive pediatric papillary thyroid carcinoma (PTC) often presents with regional nodal or distant disease, but factors explaining heterogeneity within fusion-positive tumors are incompletely defined.

**Objective:**

To determine whether age, fusion group, or sex is associated with baseline N1b and/or M1 disease in fusion-positive pediatric and young adult PTC.

**Design:**

Retrospective analysis of the international PEDIMAP cohort.

**Setting:**

Multi-institutional pediatric and young adult thyroid carcinoma cohort.

**Patients:**

Patients aged 0-25 years with PTC, documented somatic kinase fusion, and available fusion-partner information.

**Intervention(s):**

None.

**Main Outcome Measure(s):**

Advanced baseline presentation, defined as AJCC N1b and/or M1 disease. Associations were tested using Firth penalized logistic regression.

**Results:**

Of 101 kinase fusion-positive PTCs identified among 646 PTCs, 2 rearranged during transfection (RET)-fusion cases were excluded because fusion-partner information was unavailable, yielding 99 fusion-positive tumors with known fusion partners: RET, n = 52; neurotrophic receptor tyrosine kinase (NTRK), n = 28; and other non-RET/NTRK kinase fusions, n = 19. Among 95 patients with evaluable N- and M-stage, 64 (67.4%) had N1b and/or M1 disease. Age 0-14 years was associated with higher odds of advanced presentation than age 15-25 years (adjusted OR, 4.67; 95% CI, 1.84-12.75; *P* = .001). No significant difference in advanced presentation was found between *NTRK* and *RET* fusions (adjusted OR, 0.83; 95% CI, 0.29-2.50; *P* = .739).

**Conclusion:**

Within fusion-positive pediatric and young adult PTC, younger age was associated with greater baseline disease extent, whereas no significant difference in N1b/M1 presentation was found between *RET* and *NTRK*-fusion groups. These findings support careful baseline assessment of regional and distant disease in younger fusion-positive patients.

Papillary thyroid carcinoma (PTC) in children, adolescents, and young adults differs biologically and clinically from adult PTC (1). Younger patients more often present with regional nodal disease as well as pulmonary metastases. Molecularly, these tumors are enriched for chromosomal rearrangements, most commonly kinase fusions, rather than pathogenic single nucleotide variants (2). Among these alterations, rearranged during transfection (RET) and neurotrophic receptor tyrosine kinase (NTRK) fusions are among the most common oncogenic drivers in pediatric PTC consistently linked to high-burden disease at diagnosis, including lateral neck nodal metastasis and distant metastatic spread (3, 4).

While prior studies have shown that fusion-positive pediatric PTC is often advanced at presentation (5), it remains unclear whether variation within fusion-positive disease is driven primarily by fusion group, specific fusion partner, histologic phenotype, developmental age, or their interaction. This is clinically relevant because *RET* and *NTRK* fusions are biologically distinct, recurrent partners such as *CCDC6::RET*, *NCOA4::RET*, and *ETV6::NTRK3* may have different morphologic associations, and younger age is itself linked to more advanced pediatric thyroid carcinoma (3, 4, 6-9).

This study examines these questions in a pediatric and young adult fusion-positive PTC cohort with known fusion partners. Tumors were grouped by clinically interpretable fusion group, recurrent and rare partners were documented, and baseline advanced presentation was defined using a AJCC N1b and/or M1 endpoint. The composite endpoint was further separated into N1b + M1, N1b-only, M1-only, and neither categories.

We hypothesized that developmental age would remain a key determinant of baseline disease extent within this molecularly enriched cohort. By integrating age, fusion group, partner-level annotation, histologic phenotype, and N1b and M1 category designation, the study evaluates how age-specific molecular information is critical to anticipating baseline staging in fusion-positive pediatric and young adult PTC.

## Materials and methods

### Study design and cohort source

We performed a retrospective analysis of the international PEDIMAP cohort, a multi-institutional REDCap database (10) that records clinical, cytologic, histopathologic, molecular, treatment, and follow-up data for pediatric and young adult patients with thyroid nodules or thyroid carcinoma. This analysis included patients aged 0-25 years with PTC and a documented somatic kinase fusion and associated fusion partner.

Of 646 PTCs in our REDCap cohort, 183 had an identified somatic molecular alteration and 101 were classified as kinase fusion-positive before partner-level review. Two *RET*-fusion cases were excluded because the fusion partner could not be recovered. The final analytic cohort therefore included 99 fusion-positive PTCs with known fusion partners.

The PEDIMAP protocol was reviewed by the Committees for the Protection of Human Subjects at the Children's Hospital of Philadelphia and determined to be exempt under CHOP IRB #23-021615. A waiver of HIPAA authorization was granted, and all analyses were performed using deidentified data.

### Data collection and variable definitions

Clinical, demographic, pathologic, molecular, treatment, and follow-up data were abstracted from the PEDIMAP cohort. Clinical variables included age at diagnosis, sex, year of diagnosis, surgical approach, radioactive iodine treatment, and follow-up duration, calculated in years from surgery to the most recent clinical assessment. Age at diagnosis was categorized using the predefined PEDIMAP developmental age strata: 0-8, 9-14, 15-18, and 19-25 years. For regression modeling, age was collapsed a priori into 0-14 vs 15-25 years (11-14).

Preoperative cytology, when available, was classified according to the third edition of the Bethesda System for Reporting Thyroid Cytopathology (TBSRTC) (15) and retained as a descriptive baseline variable. Histopathologic subtype, American Joint Committee on Cancer (AJCC) Lymph Node (N) stage and distant metastasis (M) stage, and somatic molecular findings were abstracted from institutional pathology and molecular profiling reports.

The primary study endpoint was baseline advanced anatomic presentation, defined by AJCC N1b and/or M1 disease at diagnosis (16). Because lateral neck nodal metastasis and distant metastasis are separate disease sites that can occur alone or together, patients were assigned to 1 of 4 mutually exclusive baseline categories: both N1b and M1, N1b-only, M1-only, or neither N1b nor M1. Patients with unknown or missing M-stage (Mx or blank) were excluded from analyses that required M-stage information, including the N1b/M1 composite and overlap categories.

Disease status at the last available follow-up was recorded as an exploratory variable using response-to-therapy/dynamic risk stratification categories based on the 2025 American Thyroid Association adult differentiated thyroid carcinoma guidelines. Patients were classified as having an excellent response, indeterminate response, biochemical incomplete response, structural incomplete response, or unknown/unclassifiable disease status. Classification incorporated imaging findings, serum thyroglobulin and thyroglobulin-antibody status, extent of thyroid surgery, and whether radioactive iodine had been administered before the assessment. This variable was used descriptively to relate baseline anatomic presentation to later disease status and was not used as the primary outcome (17).

### Somatic molecular profiling and fusion classification

Somatic molecular profiling was performed at each participating institution as part of routine clinical care, using commercial or validated in-house molecular platforms capable of detecting clinically relevant thyroid carcinoma driver alterations, including gene fusions. Because molecular testing spanned multiple institutions, the specific assay used was not uniform across cases. When available, testing included fusion-capable next-generation sequencing panels, RNA-based sequencing, or other validated molecular assays able to detect kinase fusions.

Among 646 PTCs in PEDIMAP, 183 had an identified somatic molecular alteration and 101 were classified as kinase fusion-positive PTC before fusion-partner review. Two *RET*-fusion cases were excluded because the fusion partner was unavailable, yielding a final analytic cohort of 99 fusion-positive PTCs with known fusion partners.

To support the clinically interpretable analysis, tumors were classified into 3 mutually exclusive groups: *RET* fusions, *NTRK* fusions, and other non-*RET/NTRK* kinase fusions. *NTRK1* and *NTRK3* fusions were grouped within the NTRK group. The other non-*RET/NTRK* kinase group included *ALK*, *BRAF*, and *MET* fusions. The final fusion-group distribution was *RET* fusion, n = 52; *NTRK* fusion, n = 28; and other non-*RET/NTRK* kinase fusion, n = 19.

### Statistical analysis

Cohort characteristics were summarized using descriptive statistics. Categorical variables were reported as counts and percentages, and continuous variables as medians with interquartile ranges. For each variable, percentages were calculated only among patients with available data for that specific variable; patients with missing data for that variable were not included in the denominator.

The primary analysis tested which baseline factors were associated with advanced anatomic presentation at diagnosis, defined as AJCC N1b and/or M1 disease. Because the cohort was relatively small and some comparison groups had few patients, we used Firth penalized logistic regression, a regression method designed to reduce bias in small or sparse datasets (18). The model included 3 prespecified variables: fusion group, age group, and sex. Fusion group was compared as *RET*, *NTRK*, or other non-*RET/NTRK* kinase fusion; age was compared as 0-14 vs 15-25 years; and sex as male vs female.

For each comparison, 1 group was used as the reference group: *RET* fusion for fusion group, age 15-25 years for age group, and female sex for sex. Results are reported as adjusted odds ratios with 95% confidence intervals and *P* values. In practical terms, these estimates describe whether each factor was associated with higher or lower odds of advanced presentation after accounting for the other variables in the model.

We also performed several prespecified sensitivity analyses to test whether the main findings were consistent under different assumptions. These included a model using N1b disease alone as the outcome, a model adjusted for diagnosis era, a model adjusted for diffuse-sclerosing histology, a model excluding diffuse-sclerosing PTC, and a model restricted to the 2 largest fusion groups, *RET* and *NTRK*. ATA/disease-status response at the last follow-up was summarized descriptively by baseline N1b/M1 category and was not used as a primary outcome.

Missing values were not imputed. Each analysis included only patients with the data needed for that specific variable or model. Analyses were performed in R version 4.6.0 (19) using the logistf package for Firth penalized logistic regression (20).

Figures were generated in Python 3.13.5 using matplotlib (21) and NumPy (22).

## Results

### Cohort assembly and baseline characteristics

**Figure 1:**
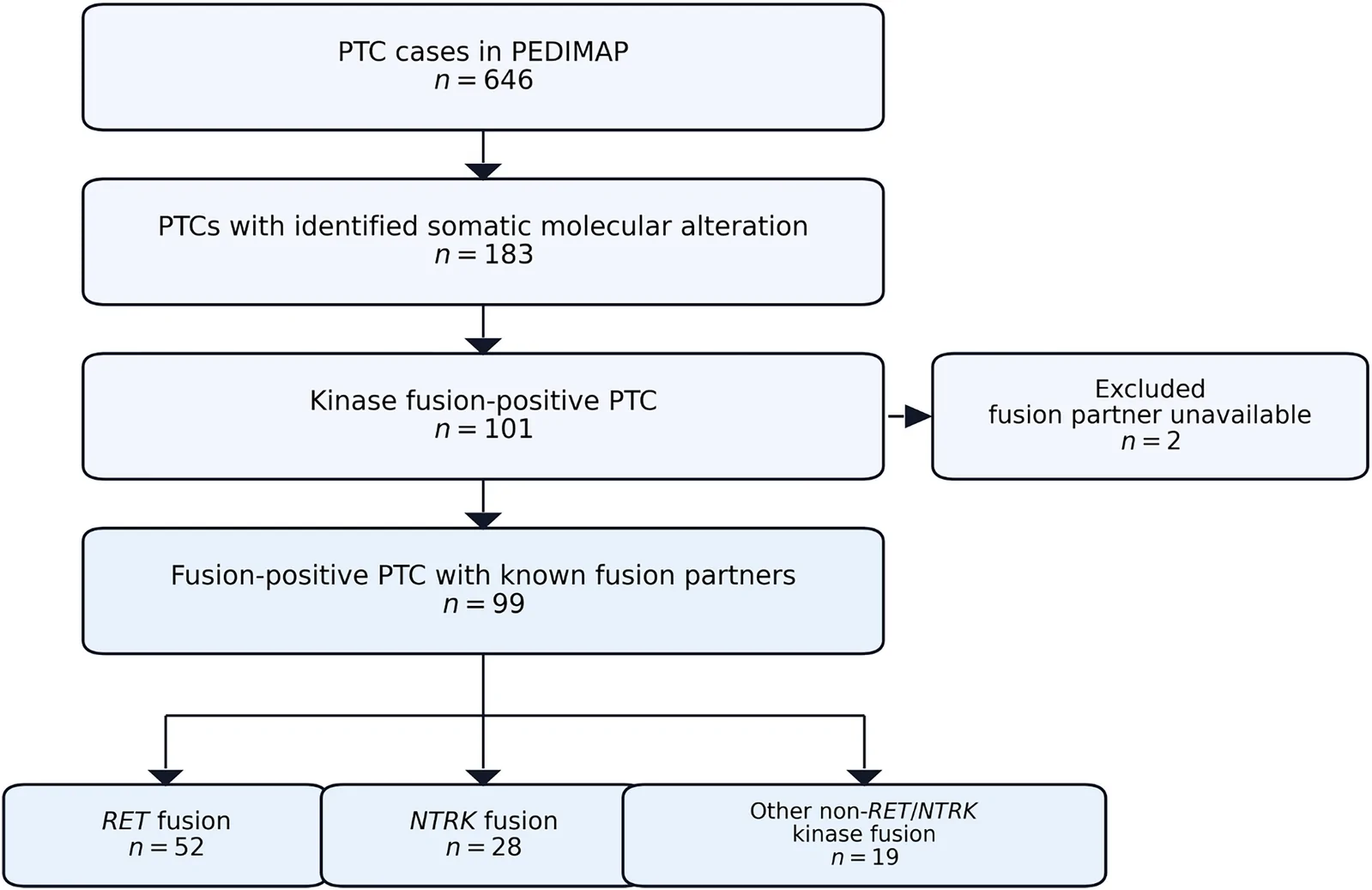
Cohort flow diagram. Flow diagram showing assembly of the fusion-positive PTC cohort from PEDIMAP. Among 646 PTC cases, 183 had an identified somatic molecular alteration and 101 were kinase fusion-positive. Two cases were excluded because fusion-partner information was unavailable, yielding 99 fusion-positive PTCs with known fusion partners: *RET* fusion, n = 52; *NTRK* fusion, n = 28; and other non-*RET*/*NTRK* kinase fusion, n = 19.

Among 646 PTCs in the REDCap cohort, 183 had an identified somatic molecular alteration and 101 were classified as kinase fusion-positive PTC before fusion-partner review. Two RET-fusion cases were excluded because fusion-partner information was unavailable, yielding a final analytic cohort of 99 fusion-positive PTCs with known fusion partners (Fig. 1).

The median year of diagnosis was 2019 (IQR, 2016.5-2021.0). The cohort was separated into age bands: 6/99 patients (6.1%) were aged 0-8 years, 51/99 (51.5%) were aged 9-14 years, 38/99 (38.4%) were aged 15-18 years, and 4/99 (4.0%) were aged 19-25 years. For regression modeling, the 2 youngest and 2 oldest cohorts were combined: 57/99 patients (57.6%) were grouped as 0-14 years and 42/99 (42.4%) as 15-25 years. Female patients predominated, comprising 77/99 patients (77.8%) compared with 22/99 (22.2%) male patients.

Surgical treatment was nearly uniform: 98/99 patients (99.0%) underwent total thyroidectomy and 1/99 (1.0%) underwent lobectomy. RAI status was available for 97/99 patients, of whom 80/97 (82.5%) received RAI. Follow-up duration was available for 84/99 patients, with a median of 3.03 years (IQR, 1.54-5.42). ATA/disease-status response at last follow-up was available for 63/99 patients (63.6%) (Table 1).

**Table 1:** Cohort characteristics of fusion-positive PTC cohort with known fusion partners, n = 99

Characteristic	Value
Diagnosis year	2019, IQR 2016.5-2021.0
Age band at diagnosis	
0-8 years	6/99 (6.1%)
9-14 years	51/99 (51.5%)
15-18 years	38/99 (38.4%)
19-25 years	4/99 (4.0%)
Model age group	
0-14 years	57/99 (57.6%)
15-25 years	42/99 (42.4%)
Sex	
Male	22/99 (22.2%)
Female	77/99 (77.8%)
Surgical approach	
Lobectomy	1/99 (1.0%)
Total thyroidectomy	98/99 (99.0%)
Histologic subtype	
Classic PTC	55/97 (56.7%)
Diffuse-sclerosing PTC	22/97 (22.7%)
Infiltrative follicular-patterned PTC	14/97 (14.4%)
Other/rare/mixed PTC	6/97 (6.2%)
Fusion group	
RET	52/99 (52.5%)
NTRK	28/99 (28.3%)
Other non-RET/NTRK fusion	19/99 (19.2%)
Fusion-partner group	
CCDC6::RET	26/99 (26.3%)
NCOA4::RET	11/99 (11.1%)
Other RET fusions	15/99 (15.2%)
ETV6::NTRK3	18/99 (18.2%)
Other NTRK fusions	10/99 (10.1%)
Other non-RET/NTRK kinase fusions	19/99 (19.2%)
Preoperative Bethesda v3 cytology available	89/99 (89.9%)
Bethesda II/benign	1/89 (1.1%)
Bethesda III/IV/indeterminate	7/89 (7.9%)
Bethesda V/VI/suspicious or malignant	81/89 (91.0%)
Missing Bethesda v3 cytology	10/99 (10.1%)
RAI treatment available	97/99 (98.0%)
RAI yes	80/97 (82.5%)
RAI no	17/97 (17.5%)
Missing RAI status	2/99 (2.0%)
Follow-up duration available	84/99 (84.8%)
Follow-up duration, years	3.03, IQR 1.54-5.42
ATA 2025/disease-status response available	63/99 (63.6%)

Percentages use the full cohort denominator unless a nonmissing denominator is shown. Histologic subtype percentages are calculated among the 97 tumors with documented histologic subtype; 2 tumors lacked documented histologic subtype. Bethesda percentages are calculated among the 89 cases with available Bethesda v3 cytology. RAI percentages are calculated among the 97 cases with available RAI status.

### Fusion and partner distribution

*RET* fusions were the most common alteration, occurring in 52/99 patients (52.5%). *NTRK* fusions were identified in 28/99 patients (28.3%), and other non-*RET/NTRK* kinase fusions were identified in 19/99 patients (19.2%). The most frequent individual fusion partners were *CCDC6::RET* (26/99, 26.3%), *ETV6::NTRK3* (18/99, 18.2%), and *NCOA4::RET* (11/99, 11.1%). Less frequent partners included additional *RET* and *NTRK* fusions, as well as other kinase fusions involving *ALK* (n = 9), *BRAF* (n = 6), and *MET* (n = 4).

Preoperative cytologic diagnosis as per TBSRTC was available for 89/99 patients (89.9%). Among these cytology-evaluable cases, 81/89 (91.0%) were classified as Bethesda V/VI, 7/89 (7.9%) as Bethesda III/IV, and 1/89 (1.1%) as Bethesda II. The Bethesda III/IV subset included 2 Bethesda III/AUS and 5 Bethesda IV/follicular neoplasm cases.

**Figure 2:**
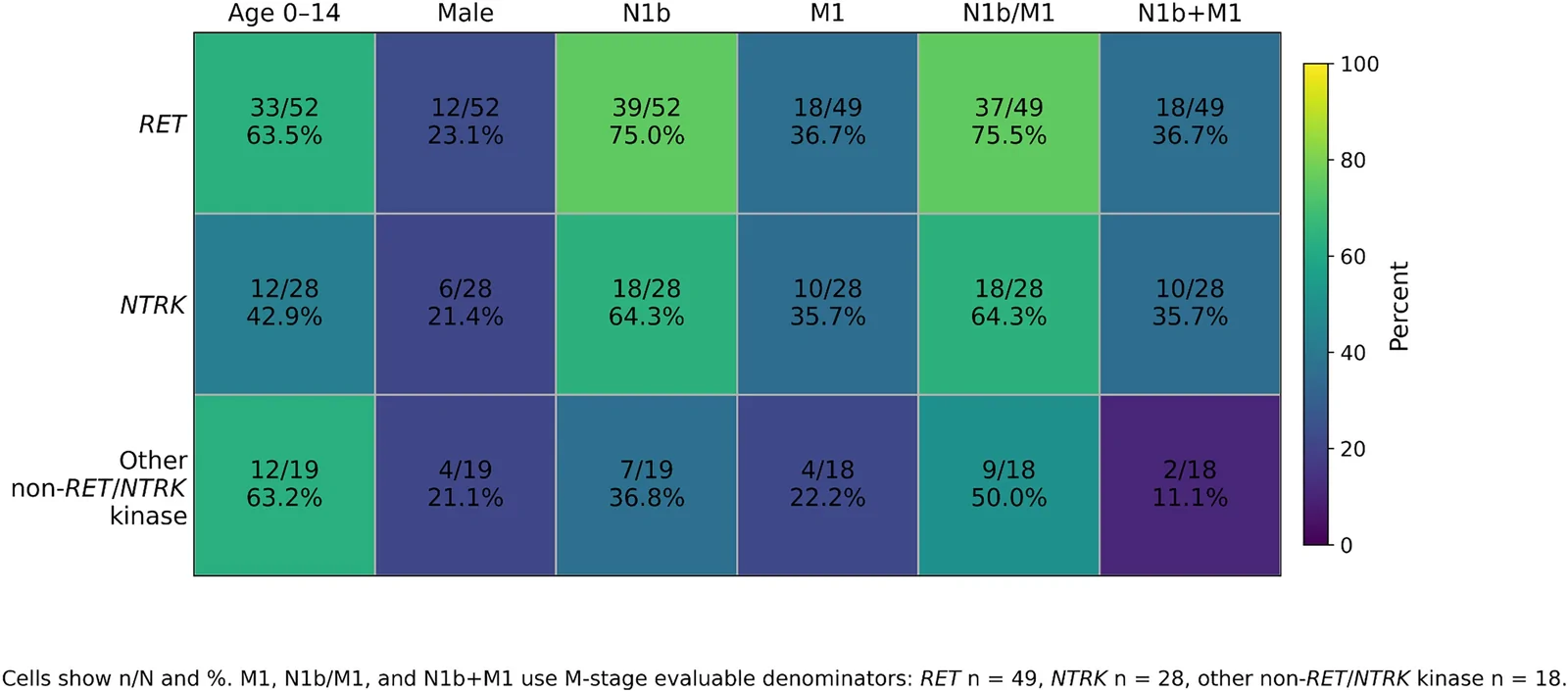
Fusion group clinical heatmap. Clinical features by fusion group in the fusion-positive PTC cohort. Cells show n/N and percentage for age 0-14 years, male sex, N1b disease, M1 disease, complete-case N1b and/or M1 presentation, and combined N1b + M1 disease. M-stage-dependent columns use M-stage evaluable denominators: *RET* n = 49, *NTRK* n = 28, and other non-*RET*/*NTRK* kinase fusion n = 18.

Clinical features differed somewhat by fusion groups, but the patterns overlapped considerably (Fig. 2).

Patients aged 0-14 years accounted for 33/52 *RET*-fusion tumors (63.5%), 12/28 *NTRK*-fusion tumors (42.9%), and 12/19 other non-*RET/NTRK* kinase-fusion tumors (63.2%). Male sex was similarly distributed across the 3 fusion groups. N1b disease was most common in the *RET*-fusion group, occurring in 39/52 patients (75.0%). It was also frequent in the *NTRK*-fusion group, occurring in 18/28 patients (64.3%), but less common in the other non-*RET/NTRK* kinase-fusion group, occurring in 7/19 patients (36.8%). Among patients with evaluable M-stage, M1 disease was present in 18/49 *RET*-fusion patients (36.7%), 10/28 *NTRK*-fusion patients (35.7%), and 4/18 patients with other non-*RET/NTRK* kinase fusions (22.2%).

**Figure 3:**
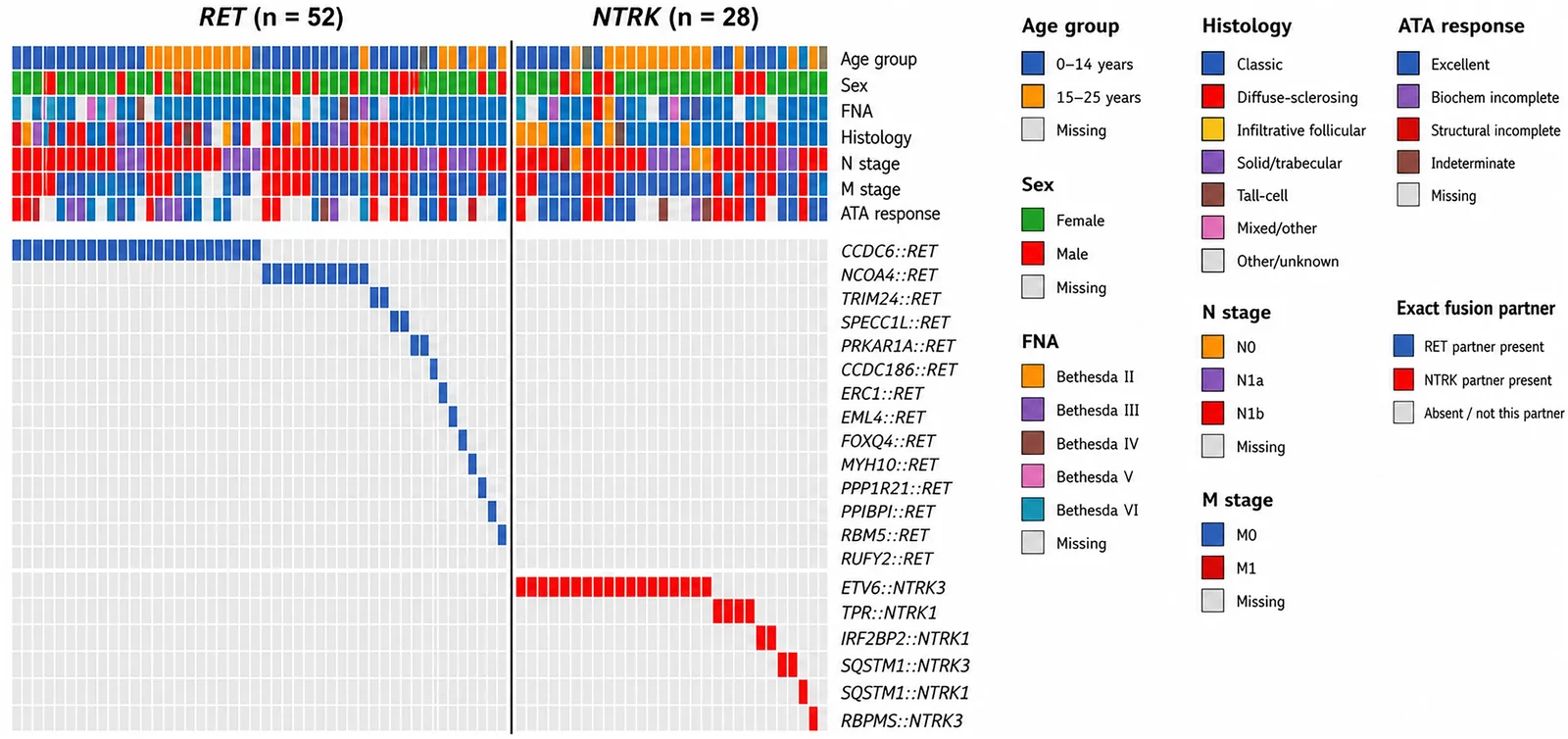
Case-level clinicopathologic matrix of the *RET*/*NTRK* fusion-positive subcohort. Case-level matrix of *RET*- and *NTRK*-fusion-positive PTCs with specific fusion-partner assignment. Each column represents 1 case. Upper annotation tracks show age group, sex, preoperative FNA category, histologic subtype, N-stage, M-stage, and ATA/disease-status response at last follow-up. Lower rows indicate the specific fusion partner within each fusion group. Gray denotes missing, unavailable, or nonapplicable data.

Using the complete-case definition, advanced N1b/M1 presentation was seen in 37/49 *RET*-fusion patients (75.5%), 18/28 *NTRK*-fusion patients (64.3%), and 9/18 patients with other non-*RET/NTRK* kinase fusions (50.0%). Combined N1b + M1 disease followed a similar pattern but was less frequent in the other non-*RET/NTRK* kinase-fusion group. It occurred in 18/49 *RET*-fusion patients (36.7%), 10/28 *NTRK*-fusion patients (35.7%), and 2/18 patients with other non-*RET/NTRK* kinase fusions (11.1%). A case-level matrix of the *RET/NTRK* fusion-positive subcohort is shown in Fig. 3.

### Baseline anatomic presentation

AJCC N stage was available for all 99 patients. N1b disease was frequent, occurring in 64/99 patients (64.6%). The remaining patients had either N0a disease (12/99, 12.1%) or N1a disease (23/99, 23.2%).

AJCC M-stage was evaluable in 95/99 patients after Mx and blank M-stage values were excluded. Among these patients, 32/95 (33.7%) had M1 disease and 63/95 (66.3%) had M0 disease. Using the complete-case definition, which required evaluable M-stage, advanced baseline presentation was defined as N1b and/or M1 disease. This was present in 64/95 patients (67.4%); the remaining 31/95 patients (32.6%) had neither N1b nor M1 disease.

**Figure 4:**
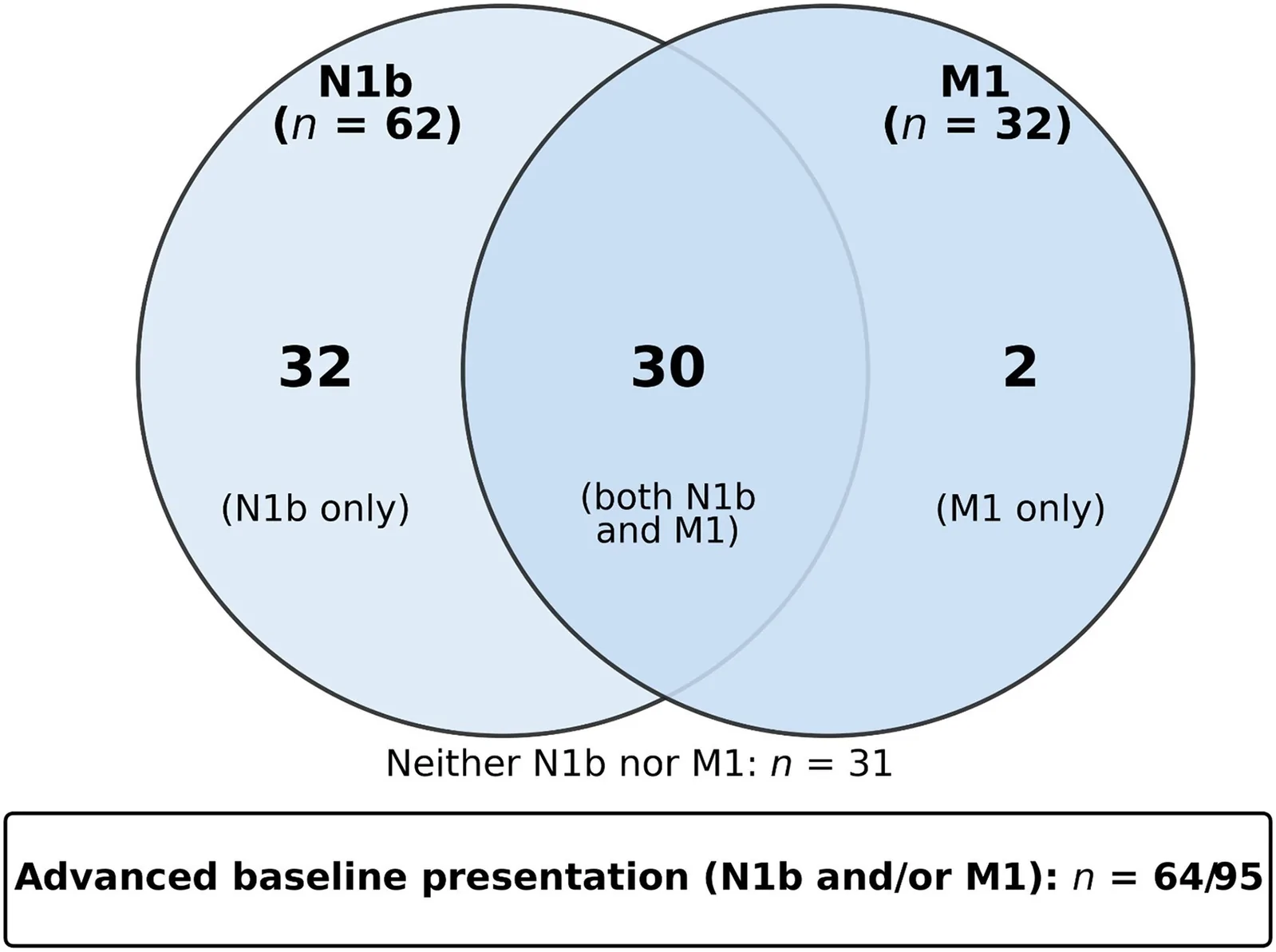
N1b/M1 overlap. Overlap between lateral neck nodal disease and distant metastasis among 95 patients with evaluable N- and M-stage. N1b disease was present in 62 patients and M1 disease in 32 patients; 30 patients had both N1b and M1 disease, 32 had N1b-only disease, 2 had M1-only disease, and 31 had neither N1b nor M1 disease. Complete-case advanced baseline presentation, defined as N1b and/or M1 disease, was present in 64/95 patients.

The N1b/M1 overlap showed 4 presentation patterns: 30/95 patients (31.6%) had both N1b and M1 disease, 32/95 (33.7%) had N1b-only disease, 2/95 (2.1%) had M1-only disease, and 31/95 (32.6%) had neither N1b nor M1 disease (Table 2; Fig. 4).

**Table 2:** Baseline anatomic presentation in the fusion-positive PTC cohort with known fusion partners, n = 99

Characteristic	Value
AJCC N stage, n = 99	
N0a	12/99 (12.1%)
N1a	23/99 (23.2%)
N1b	64/99 (64.6%)
AJCC M-stage, evaluable n = 95	
M0	63/95 (66.3%)
M1	32/95 (33.7%)
Mx	2/99 excluded
Missing/blank M-stage	2/99 excluded
Total M-stage excluded	4/99 (4.0%)
Complete-case N1b/M1 overlap, n = 95	
N1b among complete N/M cases	62/95 (65.3%)
M1 among complete N/M cases	32/95 (33.7%)
Both N1b and M1	30/95 (31.6%)
N1b-only	32/95 (33.7%)
M1-only	2/95 (2.1%)
Neither N1b nor M1	31/95 (32.6%)
Composite endpoint, complete-case classification	
Endpoint evaluable	95/99 (96.0%)
Advanced presentation, N1b and/or M1	64/95 (67.4%)
Not advanced, no N1b and confirmed M0	31/95 (32.6%)

AJCC N- and M-stage are separate REDCap variables. Under the complete-case rule, all Mx/blank M-stage cases are excluded from M-stage percentages and from derived N1b/M1 categories. Therefore, the overlap equation is: 62 + 32 − 30 = 64 advanced cases among 95 evaluable patients.

### Primary model and sensitivity analyses

The primary Firth penalized logistic regression model examined factors associated with advanced baseline presentation, defined as N1b and/or M1 disease. This analysis included 95 patients with evaluable N- and M-stage. Of these, 64 patients had advanced presentation and 31 did not. The model adjusted for fusion group, age group, and sex.

**Figure 5:**
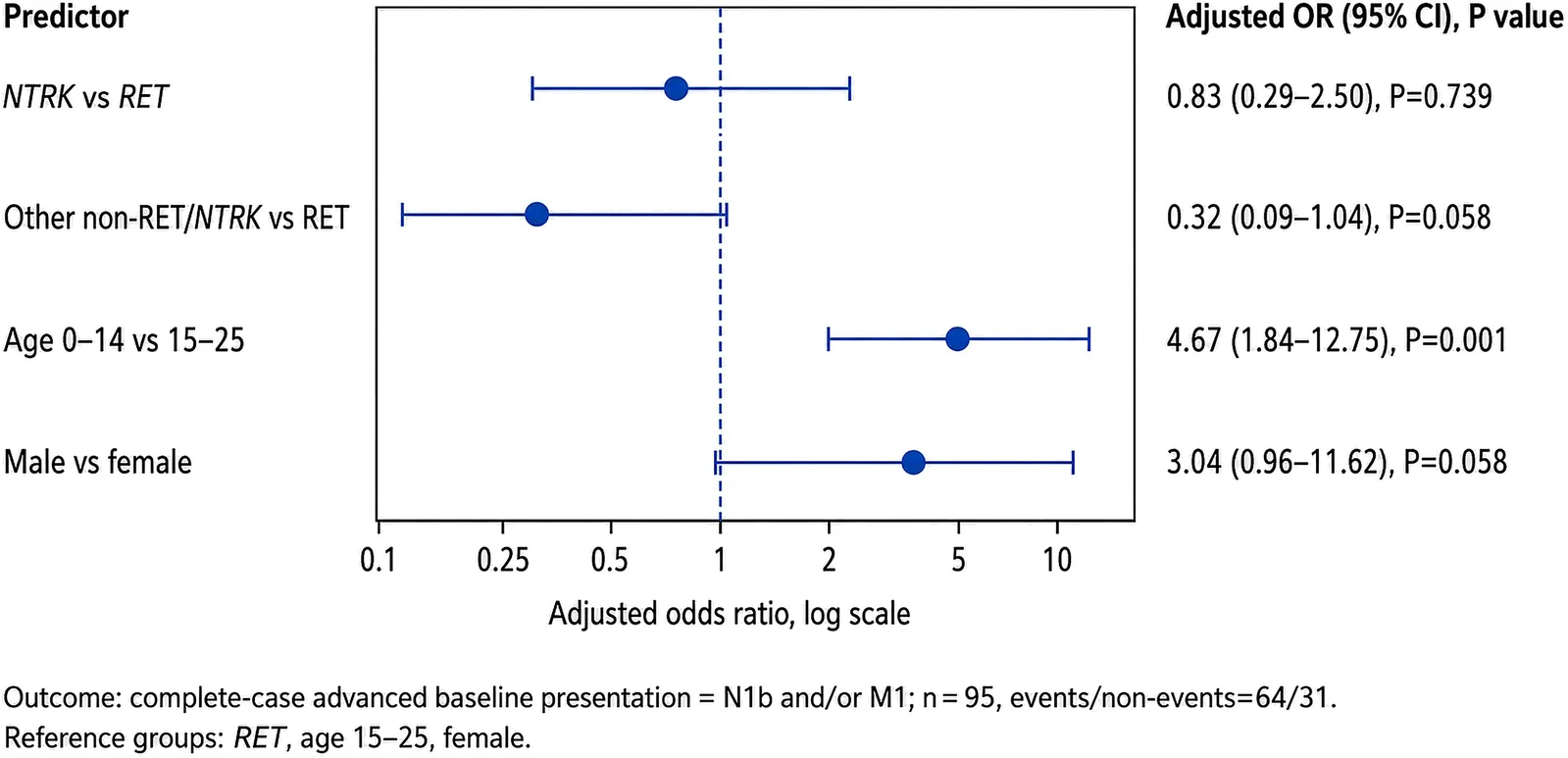
Primary Firth penalized logistic regression model for advanced baseline presentation. Forest plot showing adjusted odds ratios and 95% confidence intervals from the primary Firth penalized logistic regression model for complete-case advanced baseline presentation, defined as N1b and/or M1 disease. The model included fusion group, age group, and sex. Reference groups were *RET* fusion, age 15-25 years, and female sex. The vertical dashed line indicates an odds ratio of 1.

Age 0-14 years was associated with higher odds of advanced N1b/M1 presentation. Patients aged 0-14 years had significantly higher odds of advanced baseline metastatic presentation than patients aged 15-25 years (adjusted OR, 4.67; 95% CI, 1.84-12.75; *P* = .001). Male sex was associated with higher estimated odds, but this result did not reach conventional statistical significance (adjusted OR, 3.04; 95% CI, 0.96-11.62; *P* = .058). Fusion group did not separate patients as clearly. Compared with *RET* fusion, *NTRK* fusion showed no significant difference in advanced presentation (adjusted OR, 0.83; 95% CI, 0.29-2.50; *P* = .739). Other non-*RET/NTRK* kinase fusions showed lower estimated odds than *RET* fusion, but this difference also did not reach conventional statistical significance (adjusted OR, 0.32; 95% CI, 0.09-1.04; *P* = .058) (Table 3; Fig. 5).

**Table 3:** Primary and sensitivity models for baseline advanced presentation

Model	Method	n	Events/non-events	NTRK vs RET	Other non-RET/NTRK vs RET	Age 0-14 vs 15-25	Male vs female	Additional term/comment
Primary model: advanced N1b/M1	Firth logistic	95	64/31	0.83 (0.29-2.50), *P* = .739	0.32 (0.09-1.04), *P* = .058	4.67 (1.84-12.75), *P* = .001	3.04 (0.96-11.62), *P* = .058	Primary complete-case model
N1b-only sensitivity	Firth logistic	99	64/35	0.75 (0.27-2.18), *P* = .597	0.17 (0.05-0.54), *P* = .002	3.76 (1.51-10.01), *P* = .004	3.49 (1.10-13.64), *P* = .034	Outcome = N1b-only
Era-adjusted sensitivity	Firth logistic	95	64/31	0.84 (0.29-2.50), *P* = .745	0.33 (0.10-1.06), *P* = .062	4.54 (1.79-12.37), *P* = .001	2.99 (0.95-11.34), *P* = .061	Diagnosis era 2020 + vs ≤2019: 0.91 (0.36-2.32), *P* = .837
Diffuse-sclerosing adjusted sensitivity	Firth logistic	95	64/31	1.30 (0.41-4.29), *P* = .660	0.45 (0.13-1.53), *P* = .202	4.94 (1.85-14.47), *P* = .001	2.56 (0.75-10.43), *P* = .139	Diffuse-sclerosing PTC: 9.02 (1.84-90.98), *P* = .004
Diffuse-sclerosing excluded sensitivity	Firth logistic	73	43/30	1.20 (0.38-3.96), *P* = .761	0.41 (0.11-1.43), *P* = .160	4.54 (1.65-13.63), *P* = .003	2.34 (0.66-9.80), *P* = .195	Excludes diffuse-sclerosing PTC
RET/NTRK-only sensitivity	Firth logistic	77	55/22	0.83 (0.27-2.62), *P* = .743	—	5.20 (1.76-16.79), *P* = .003	8.39 (1.75-83.35), *P* = .005	Restricted to RET and NTRK fusion families

Outcome for primary model: advanced baseline presentation = N1b and/or M1. Primary model uses the complete-case endpoint, excluding all 4 Mx/blank M-stage cases. Values are adjusted odds ratios with 95% profile-likelihood confidence intervals and penalized-likelihood-ratio *P* values from Firth logistic regression. The primary model includes fusion group, age group, and sex. The complete-case advanced-presentation endpoint excludes all 4 patients with Mx or blank M-stage, yielding 95 evaluable patients.

The sensitivity analyses were consistent with the association between younger age and advanced baseline presentation. Age 0-14 years remained associated with N1b disease in the N1b-only model and with advanced N1b/M1 presentation after adjustment for diagnosis era. The age association also persisted after adjustment for diffuse-sclerosing histology, after excluding diffuse-sclerosing PTC, and in the model restricted to *RET*- and *NTRK*-fusion tumors. Male sex was associated with N1b disease in the N1b-only model and with advanced N1b/M1 presentation in the *RET/NTRK*-only model. Other non-*RET/NTRK* kinase fusions were associated with lower odds of N1b disease in the N1b-only model. No significant difference was found between *NTRK* and *RET* fusion in either the primary model or the *RET/NTRK*-restricted model (Table 3).

### ATA 2025/disease-status response by N1b/M1 category

ATA 2025/disease-status response was assessed according to the baseline N1b/M1 categories. Of the 95 patients in the complete-case cohort, 62 had available ATA/disease-status response data (17).

Structural incomplete response was most common among patients who had both N1b and M1 disease at baseline. In this combined N1b + M1 group, 16/21 patients (76.2%) had a structural incomplete response, and 19/21 (90.5%) had a nonexcellent response. By comparison, structural incomplete response was uncommon in patients with N1b-only disease (1/20, 5.0%) and in patients with neither N1b nor M1 disease (1/19, 5.3%). The M1-only subgroup included only 2 patients, and both had an excellent response; this result should be interpreted cautiously because of the very small subgroup size (Table 4).

**Table 4:** Exploratory ATA 2025/disease-status response by baseline N1b/M1 category

Baseline anatomic category	Total n	ATA response available	Excellent response	Nonexcellent response	Structural incomplete response
N1b + M1	30	21	2/21 (9.5%)	19/21 (90.5%)	16/21 (76.2%)
N1b-only	32	20	13/20 (65.0%)	7/20 (35.0%)	1/20 (5.0%)
M1-only	2	2	2/2 (100.0%)	0/2 (0.0%)	0/2 (0.0%)
Neither N1b nor M1, confirmed M0	31	19	12/19 (63.2%)	7/19 (36.8%)	1/19 (5.3%)
Total classifiable	95	62	29/62 (46.8%)	33/62 (53.2%)	18/62 (29.0%)

Response-to-therapy/disease-status categories were assigned according to the 2025 ATA adult differentiated thyroid carcinoma dynamic risk stratification framework and analyzed descriptively. Percentages use cases with available response data as the denominator. Four patients with Mx or blank M-stage were excluded from the complete-case N1b/M1 categories; one excluded N1b/Mx-or-blank case had available response data, explaining why response availability changes from 63 in the full cohort to 62 in the complete-case cohort.

Overall, 18/62 patients (29.0%) with available response data in the complete-case cohort had a structural incomplete response. These findings suggest that the clinical interpretation of the composite N1b/M1 endpoint was driven mainly by patients with both lateral neck nodal disease and distant metastasis, rather than by patients with isolated N1b disease alone.

## Discussion

Kinase fusion-positive pediatric PTC is recognized as a clinically important subset that often presents with extensive metastatic disease. Early pediatric studies report that *RET*- and *NTRK*-rearranged tumors are larger, more often diffuse and solid, and more frequently associated with lymphatic and vascular invasion compared with *BRAF* V600E-mutant tumors (6, 7). Pekova et al similarly linked fusion-positive PTC to more advanced clinicopathologic features, including extrathyroidal extension, nodal and distant (pulmonary) metastasis, and greater use of radioactive iodine (5). Franco et al reported greater metastatic burden and lower 1-year remission in *RET/NTRK* fusion-positive pediatric differentiated thyroid carcinoma than in *BRAF*-mutant tumors (3). Building on this work, the present study focused on a narrower question: among fusion-positive pediatric and young adult PTCs, which factors best stratify baseline N1b/M1 presentation?

Consistent with prior fusion-positive pediatric PTC cohorts, advanced baseline disease was common in this study: 64/95 patients with evaluable AJCC N- and M-stage had N1b and/or M1 disease (3, 5-7). Within this fusion-positive group, developmental age was most consistently associated with baseline anatomic burden. Patients aged 0-14 years had substantially higher odds of N1b/M1 presentation than those aged 15-25 years, even after adjustment for fusion group and sex. Fusion group remained biologically relevant, but no significant difference was found between *NTRK* and *RET* fusions in the primary model, and the other non-*RET/NTRK* kinase-fusion group showed a lower point estimate that should be interpreted cautiously. These findings suggest that fusion-positive status provides the molecular setting, while developmental age helps refine the likelihood of advanced baseline disease within that setting.

Together, the PEDIMAP studies support a stepwise view of thyroid disease across the 0-25-year age range. The PEDIMAP cytology study showed that age changes the pretest and post-test probability of malignancy, that within the same cytology category there are different risks of malignancy in younger children compared with young adults (23). The PEDIMAP molecular driver study showed that *RET* and *NTRK* fusions are enriched in younger patients and are associated with less favorable ATA-defined disease status after treatment, whereas *RAS*- and *DICER1*-altered tumors have a more favorable response-to-therapy (11). The present study adds the baseline staging layer: among patients with fusion-positive PTC, developmental age was associated with the likelihood of N1b/M1 disease at diagnosis. In this framework, age informs diagnostic probability, molecular class informs biologic risk, and careful baseline assessment of regional and distant disease may be particularly relevant in younger fusion-positive patients.

*RET* and *NTRK* fusions are the main fusion groups for comparison in this cohort. In adult and adult-predominant thyroid carcinoma series, these rearrangements account for only a minority of cases. In contrast, pediatric PTC is more commonly driven by chromosomal rearrangements, including *RET* fusions-especially *CCDC6::RET* and *NCOA4::RET* (5, 8, 24, 25). In the present pediatric and young adult fusion-positive cohort, *RET* and *NTRK* fusions together accounted for 80/99 cases. This provided an opportunity to assess whether fusion group itself explained baseline N1b/M1 presentation.

*RET* was the largest fusion group and had the highest observed burden of regional and advanced disease. N1b disease was present in 39/52 *RET*-fusion tumors, N1b/M1 presentation in 37/49 *RET*-fusion tumors with evaluable M-stage, and combined N1b + M1 disease in 18/49. This pattern is consistent with *RET*-focused studies showing that *RET* fusion-positive PTC is enriched in pediatric and adolescent patients and is often associated with lymph node and distant metastases (6). In the large cohort reported by Pekova et al, *RET* fusions were more common in pediatric/adolescent PTC than in adult PTC, and *RET* fusion-positive carcinomas showed greater metastatic activity than *NTRK*-, *BRAF* V600E-, and *RAS*-altered carcinomas (6). Our cohort shows a similar high-burden, *RET*-enriched pattern, but within a fully fusion-positive comparison rather than a broad driver-class comparison.

*NTRK* fusions were the second largest fusion group and showed observed baseline overlap with *RET* fusions. Previous childhood PTC studies have linked *ETV6::NTRK3* to follicular-patterned morphology and, in some cohorts, to a less aggressive profile than *RET/PTC3* (26). However, *NTRK*-positive tumors in the present cohort showed considerable disease burden at diagnosis. N1b disease was present in 18/28 *NTRK*-fusion tumors, N1b/M1 presentation in 18/28, and M1 disease in 10/28. The frequency of M1 disease was numerically close to that observed in *RET*-fusion tumors among patients with evaluable M-stage, and no significant difference in advanced N1b/M1 presentation was found between *NTRK* and *RET* fusion in the adjusted primary model. Thus, although *RET* and *NTRK* fusions are biologically distinct, the present data do not support separating them into different baseline N1b/M1 risk groups based on fusion group alone.

Morphology patterns further support this interpretation. Diffuse-sclerosing PTC has been closely linked to *RET* rearrangements, and fusion-related thyroid carcinoma series show that DSPTC morphology is enriched in *RET*-fusion tumors, with *NTRK1*- or *BRAF*-fusion DSPTC occurring less frequently (24). In this cohort, diffuse-sclerosing histology was associated with advanced presentation, but the association between age 0 and 14 years and advanced presentation remained after DSPTC cases were excluded.

These findings also clarify how the N1b/M1 endpoint should be interpreted in pediatric risk assessment. Current pediatric thyroid carcinoma guidelines emphasize regional nodal disease and distant metastasis for initial risk stratification and follow-up planning, but they do not yet include a fusion-positive, age-stratified model for predicting advanced presentation (27, 28). In this cohort, patients aged 0-14 years were the fusion-positive subgroup most likely to present with N1b and/or M1 disease. Further separation of the endpoint showed that most M1 cases occurred with N1b disease, whereas isolated N1b disease was common but was much less often followed by structural incomplete response among patients with available ATA 2025/disease-status data. This distinction is important because cervical nodal metastasis is frequent in pediatric PTC and does not carry the same follow-up implications as concurrent distant metastasis that are associated with reduced disease-free survival (29-31). Practically, these results support careful baseline assessment of both regional and distant disease, especially in younger fusion-positive patients.

A key strength of this study is its focused design. To our knowledge, this is one of the largest pediatric and young adult fusion-positive PTC cohorts with known fusion partners. Rather than repeating a broad comparison of fusion-positive and mutation-driven tumors, this study examines clinically meaningful variation within fusion-positive disease. This approach allowed fusion group, recurrent partner distribution, developmental age, histologic phenotype, and N1b/M1 presentation to be evaluated as distinct but related layers of baseline disease data. The endpoint is clinically relevant because N1b and M1 are staging features that directly shape initial assessment of disease extent and subsequent risk stratification.

Several limitations should be acknowledged. Although this is a large cohort for fusion-positive pediatric and young adult PTC, rare fusion-partner subsets remain small. *NTRK1* and *NTRK3* fusions arise from distinct chromosomal loci; fusion partners were recorded individually, but *NTRK1* and *NTRK3* cases were grouped under *NTRK* for analysis. ATA/disease-status response was available only for a subset of patients and was analyzed descriptively. The retrospective multi-institutional design introduced expected variation in molecular testing platforms, imaging assessment, treatment decisions, radioactive iodine use, and follow-up duration. Therefore, the strongest conclusions relate to age- and fusion-group-level baseline staging, whereas rare partner-specific findings and follow-up associations should be considered descriptive.

In conclusion, fusion-positive pediatric and young adult PTC often presents with advanced baseline metastatic disease, but fusion group alone did not fully account for this burden. *RET* and *NTRK* fusions were the dominant high-burden groups and both showed frequent advanced baseline presentation, whereas rarer *ALK*, *BRAF*, and *MET* fusions broadened the molecular spectrum without allowing stable group-level inference. Developmental age 0-14 years showed the most consistent association with N1b/M1 presentation. Separating the composite endpoint also showed that N1b-only disease and combined N1b + M1 disease should not be treated as clinically equivalent when interpreting baseline burden and follow-up risk, including response-to-therapy interpretation. These findings support a layered staging approach for fusion-positive pediatric and young adult PTC: fusion status defines the molecular setting, fusion group and partner distribution provide biologic context; younger age may help recognize patients more likely to present with advanced disease and documented anatomic disease extent remains the foundation for initial clinical management.

## Data Availability

Deidentified data that support the findings of this study are available from the corresponding author upon reasonable request and subject to institutional approvals.
